# Blockade of VEGFR2 and Not VEGFR1 Can Limit Diet-Induced Fat Tissue Expansion: Role of Local versus Bone Marrow-Derived Endothelial Cells

**DOI:** 10.1371/journal.pone.0004974

**Published:** 2009-03-31

**Authors:** Joshua Tam, Dan G. Duda, Jean Y. Perentes, Rehan S. Quadri, Dai Fukumura, Rakesh K. Jain

**Affiliations:** 1 Edwin L. Steele Laboratory, Department of Radiation Oncology, Massachusetts General Hospital and Harvard Medical School, Boston, Massachusetts, United States of America; 2 Harvard-Massachusetts Institute of Technology Division of Health Sciences and Technology, Massachusetts Institute of Technology, Cambridge, Massachusetts, United States of America; 3 Division of Thoracic and Vascular Surgery, Centre Hospitalier Universitaire Vaudois (CHUV), Lausanne, Switzerland; Institute of Preventive Medicine, Denmark

## Abstract

**Background:**

We investigated if new vessel formation in fat involves the contribution of local tissue-derived endothelial cells (i.e., angiogenesis) or bone marrow-derived cells (BMDCs, i.e. vasculogenesis) and if antiangiogenic treatment by blockade of vascular endothelial growth factor (VEGF) receptors can prevent diet-induced obesity (DIO).

**Methodology/Principal Findings:**

We performed restorative bone marrow transplantation into wild-type mice using transgenic mice expressing green fluorescent protein (GFP) constitutively (driven by β-actin promoter) or selectively in endothelial cells (under Tie2 promoter activation) as donors. The presence of donor BMDCs in recipient mice was investigated in fat tissue vessels after DIO using *in vivo* and *ex vivo* fluorescence microscopy. We investigated the roles of VEGF receptors 1 and 2 (VEGFR1/VEGFR2) by inducing DIO in mice and treating them with blocking monoclonal antibodies. We found only marginal (less than 1%) incorporation of BMDCs in fat vessels during DIO. When angiogenesis was inhibited by blocking VEGFR2 in mice with DIO, treated mice had significantly lower body weights than control animals. In contrast, blocking VEGFR1 had no discernable effect on the weight gain during DIO.

**Conclusions/Significance:**

Formation of new vessels in fat tissues during DIO is largely due to angiogenesis rather than *de novo* vasculogenesis. Antiangiogenic treatment by blockade of VEGFR2 but not VEGFR1 may limit adipose tissue expansion.

## Introduction

Adipose tissue is one of the most highly vascularized tissues in the body, and a close functional relationship exists between fat tissue and its vasculature [1]. Adipose tissue is well-known for its angiogenic capacity, and has been used clinically to promote wound healing and revascularization [1], [2], [3]. It remains unknown if new vessel formation in fat requires blood circulating progenitors, such as bone marrow-derived cells (BMDCs, a process known as vasculogenesis). Vascular endothelial growth factor-A (VEGF-A or VEGF) is believed to be responsible for most of adipose tissue's angiogenic capacity [4], and adipogenesis is dependent on VEGF-mediated formation of new blood vessels [5].

VEGF is a master regulator of both physiologic and pathologic angiogenesis. VEGF binds to two tyrosine kinase receptors – VEGFR1 and VEGFR2. VEGFR2 activation promotes endothelial cell growth, survival, and migration, and increases vascular permeability [6]. VEGFR1 was previously thought to be a non-signaling “decoy” receptor, but recent studies have demonstrated its involvement in pathologic angiogenesis [7] and the recruitment of BMDCs [8], including macrophages and endothelial precursor cells [9]. Whereas VEGFR2 is primarily expressed by endothelial cells, VEGFR1 is expressed by multiple cell types, including cells of the myeloid lineage (e.g., macrophages [7]). These have recently been recognized as significant contributors to adipose tissue composition and function [10], [11]. Several reports have suggested that antivascular treatments could reduce body weight in mouse models of obesity [12], [13], [14], but whether antiangiogenic therapy by blockade of VEGFR1 or VEGFR2 can achieve this effect remains unknown.

Many aspects of neovascularization during diet-induced adipose tissue expansion remain poorly understood. VEGF is up-regulated during adipogenesis [15], but there are conflicting reports regarding both local and systemic VEGF levels during obesity [16], [17], [18], [19], [20]. We have previously shown that *de novo* adipogenesis (from transplanted pre-adipocyes) and neovascularization are reciprocally regulated via a VEGFR2-mediated paracrine mechanism(s) [21]. Whether blocking VEGFR2 during DIO limits fat expansion remains unknown. Other studies have reported the involvement of VEGFR1 signaling in fat tissue formation. For example, mice deficient for placental-derived growth factor (PlGF, a specific ligand for VEGFR1) have lower body weights during the later stages of DIO. However, pharmacologic inhibition of PlGF had no apparent effect [22]. Here, we show that vasculogenesis is negligible in DIO, and that VEGFR2 (but not VEGFR1) inhibition can limit DIO in mice.

## Materials and Methods

### Animal studies

All procedures were performed according to the Public Health Service Policy on Humane Care of Laboratory Animals, and approved by the Massachusetts General Hospital Institutional Animal Care and Use Committee. Wild-type (WT) C57BL/6J and FVB mice were bred and maintained in our defined flora facility. C57BL/6J mice carrying the green fluorescent protein (GFP) driven by chicken β-actin promoter and cytomegalovirus intermediate early enhancer (Actb-GFP), and FVB mice carrying GFP driven by the endothelial-specific Tie2 promoter (Tie2-GFP) were originally purchased from the Jackson Laboratory (Bar Harbor, ME) and subsequently bred in our facility. Animals were allowed food and water *ad libitum*. For DIO experiments, both animals and food were weighed at regular intervals. Food intake was calculated from the difference between initial and final food weights, food spillage was not taken into account.

### Reagents and Dosage

Rat anti-mouse monoclonal antibodies against VEGFR1 (MF1) and VEGFR2 (DC101) were generous gifts from ImClone Systems Inc. (New York, NY). DC101 was administered i.p. at 40 mg/kg body weight every 3 days. MF1 was administered i.p. at 500 µg/mouse every 2 days. 60 kcal% fat diets were purchased from Research Diets (New Brunswick, NJ).

### Bone Marrow Transplantation

Bone marrow transplantation (BMT) was performed after characterizing the sensitivity of the C57BL/6J and FVB strains to whole-body irradiation through longitudinal studies. We used strain-specific lethal doses of radiation to ensure a high degree of chimerism. Our protocol has been validated in a previous study [23], where we demonstrated that (i) over 90% of the bone marrow-derived cells were GFP+ in BMT/Actb-GFP mice; and (ii) Tie2-GFP is homogeneously expressed by both local derived and bone marrow derived endothelial cells. Whole-body irradiation, in a single dose of 9 Gy for FVB mice and 12 Gy for C57BL/6J mice, was administered to recipient mice. The recipient mice were salvaged by i.v. injection of 5×10^6^ bone marrow cells harvested from donor mice [23]. Bone marrow reconstitution was verified by flow cytometric analyses. WT C57BL/6J receiving transplants from Actb-GFP donors are referred to as WT/Actb-GFP-BMT, and WT FVB mice receiving transplants from Tie2-GFP donors are referred to as WT/Tie2-GFP-BMT.

### Immunohistochemistry

Functional (perfused) blood vessels were labeled in anesthetized mice by i.v. perfusion staining with 0.1 ml of 1 mg/ml biotinylated *Lycopersicon esculentum* (Tomato) lectin (Vector Labs, Burlingame, CA) for 5 minutes [24]. Transcardial perfusion fixation was subsequently performed using 4% paraformaldehyde. Tissue samples were excised, dehydrated overnight at 4°C in 30% sucrose in phosphate buffered saline (PBS), embedded in OCT compound and stored at −80°C. Tissue sections (20–30 µm thick) were mounted on slides using Vectashield mounting media for fluorescence (Vector Labs). Fluorescence counterstaining was performed using the nuclear dye DAPI (Molecular Probes, Eugene, OR). Co-localization of GFP^+^ BMDCs with lectin-stained capillaries was verified and quantified by confocal microscopy. The number of GFP^+^ vessels in sections and in intravital microscopy images was counted in 6–10 regions of interest and normalized by the total number of lectin-stained (*ex vivo*) or dextran-perfused (*in vivo*) vessels. For cell size quantification, inguinal and perigonadal fat pads were excised from DIO mice at various time points following the initiation of high fat diet, fixed at 4°C overnight in 4% paraformaldehyde, then embedded in paraffin. 10 µm sections were stained with hematoxylin and eosin, and imaged with a bright field microscope. Cross-sectional areas of individual fat cells were quantified using a custom-written computer macro.

### Intravital Microscopy

To visualize adipose tissue vasculature, mammary fat pad chambers (MFPC, Perentes *et al.*, submitted) were implanted in WT/Tie2-GFP-BMT in aging (more than 18 months old) mice (n = 2) and in 20–22 weeks old female mice (fed high fat diet for 6 weeks after BMT, n = 4). *In vivo* multiphoton laser-scanning microscopy [25] was used to visualize Tie2-GFP-positive BMDCs in fat tissue vasculature in BMT recipient mice bearing MFPCs. Blood flow was visualized by intravenous injections of rhodamine-dextran MW 2,000,000.

### Statistical Analysis

Student's t-test was used to evaluate statistical significance (defined as P<0.05).

## Results

### Marginal contribution of BMDCs to vessels during fat tissue expansion

**Figure 1:**
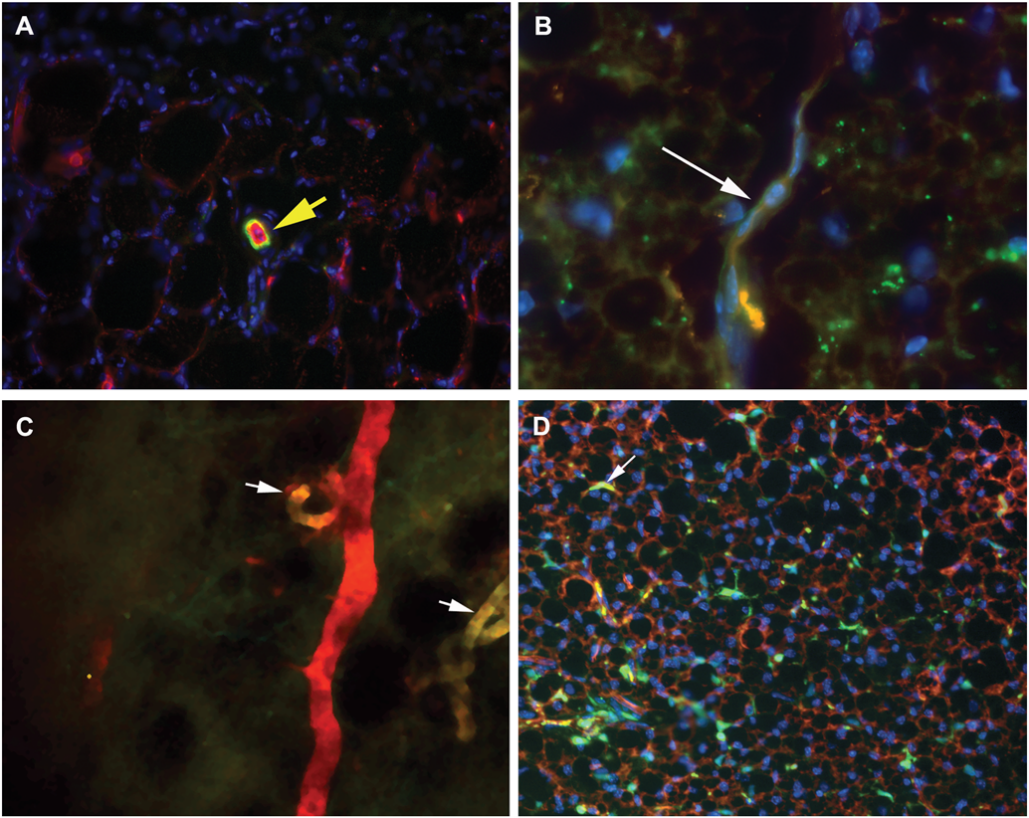
Imaging of adipose tissue vasculature in GFP transgenic mice. *A*: Incorporation of BMD-EC in mammary fat pad vasculature in WT/Tie2-GFP-BMT mice. *B*: *Ex vivo* confocal microscopy imaging of mammary fat pad in two mice with age-related obesity (∼18 months old). BMD-EC contributed to approximately 8% of the mammary fat pad vasculature. *C*: Representative image of GFP+ BMD-EC in the mammary fat pad of an obese mouse after 6 weeks on a high fat diet. BMD-EC contribution to mammary fat pad vasculature was minimal (0.8%, n = 4). Vessels were perfused with biotinylated-lectin and stained with streptavidin Texas Red (shown in red), while the nuclei were stained with DAPI (in blue, *A*, *B*). Rhodamine-dextran MW 2,000,000 was infused for vessel enhancement in *C*. *D:* Representative image of occasional GFP+ BMD-EC (arrow) in perfused mammary fat pad blood vessels in a 12 months old WT/Actb-GFP-BMT mouse. Vessels were perfused with biotinylated-lectin and stained with streptavidin Texas Red (red), while the nuclei were stained with DAPI (blue). Images are 1.72 mm across in *A*, 310 µm across in *B*, 700 µm across in *C*, 1.72 mm across in *D*.

We used bone marrow transplantation to measure the contribution of BMDCs to adipose tissue vessels in mice fed standard chow. To determine the contribution of BMDCs to fat tissue vessels, we performed lectin perfusion staining in WT/Actb-GFP-BMT mice four months post-BMT, and analyzed their abdominal fat tissue sections by confocal microscopy. We detected GFP^+^ bone marrow derived endothelial cells (BMD-EC) lining lectin-stained vessels on rare occasions (Figure 1). Of interest, in old (∼18 month-old) WT/Tie2-GFP-BMT mice that had gained weight with age, we detected BMDCs in 8.33±1.98% (n = 2) of the mammary fat pad vasculature (Figure 1). We also evaluated the contribution of BMDC to the vasculature of rapidly expanding fat tissue by feeding WT/Tie2-GFP-BMT mice a high fat diet and comparing with mice on regular diet. Blood vessels were imaged using intravital multiphoton laser-scanning microscopy, as well as by confocal microscopy in frozen sections of mammary fat pads at the end of the experiment. The high fat diet induced a significant increase in body weight after six weeks (n = 4, body weight 30.5±3.0 g vs. 20.0±0.8 g for high fat vs. regular diet-fed mice, p<0.05). The contribution of BMDCs to mammary fat vessels was estimated as the number of Tie2-GFP^+^ cells in functional (rhodamine-dextran perfused) blood vessels. BMDC contribution to fat vessels during DIO increased significantly compared to mice on regular diets, but was minimal (0.80% compared to 0.06%, p<0.05). Similar results were obtained by confocal microscopy in frozen fat tissue sections (not shown).

### Fat tissue expansion after DIO is suppressed by antibody blockade of VEGFR2, but not VEGFR1

**Figure 2:**
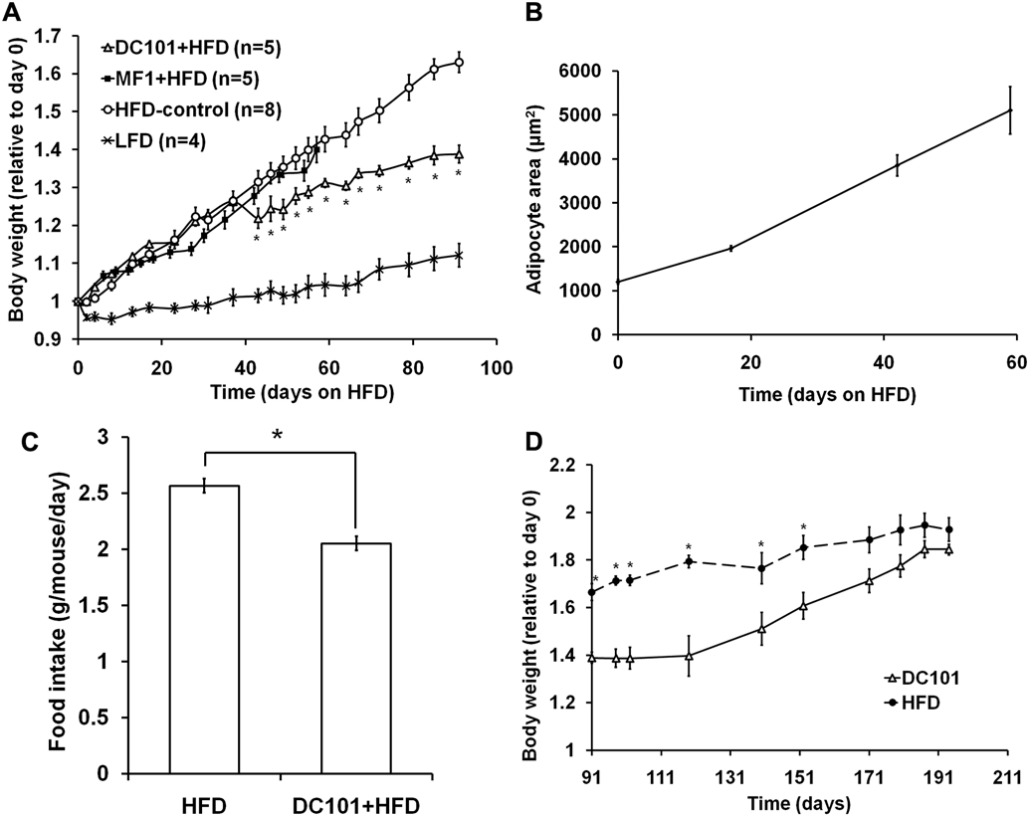
Effects of VEGFR1 and VEGFR2 blockade in mice with DIO. *A:* Body weight gain relative to weight at day 0 for mice given different diets and treatments. Male C57BL6 mice, 10–12 weeks old at time 0, were used for all groups. All diets and treatments began at day 0, at dosages and schedules as described in the Methods section. DC101+HFD: high fat diet, DC101 treatment, white triangles. MF1+HFD: high fat diet, MF1 treatment, black squares. HFD: high fat diet controls, no treatment (n = 4) or PBS treatment (n = 4), white circles. LFD: standard diet controls, crosses. *B:* Average cross-sectional area (µm^2^) of adipocytes in the perigonadal fat pad at various times after beginning of high fat diet. 3–4 mice at each time point, >300 adipocytes measured for each mouse. Adipocytes in the inguinal fat pad showed a similar trend (not shown). *C:* Food intake (g/day/kg body weight) in the DC101+HFD versus HFD groups, when the DC101+HFD group was gaining less weight (days 43–91). D: Reversibility of the effects of DC101. DC101 treatment was discontinued from day 91 onward. About two weeks after cessation of treatment, the rate of weight gain in the previously treated animals resumed at a higher rate, and their body weights eventually caught up with untreated controls. All data reported as mean±sem. Asterisks denote significant difference between DC101+HFD and HFD groups (P<0.05).

To study the role of VEGF receptors in fat tissue expansion during DIO, we monitored the body weight of high fat diet-fed mice treated with the VEGFR1-specific antibody MF1, the VEGFR2-specific antibody DC101 or with PBS, and untreated controls. The high fat diet induced rapid weight gain in control mice (Figure 2A), accompanied by a continuous increase in adipocyte size over the length of the study (Figure 2B). There was no significant difference in body weight between untreated animals and animals treated with PBS throughout the course of the experiment, therefore data from those two groups were pooled for subsequent analyses (HFD). Antiangiogenic treatment with DC101 had no significant effect on body weight during the first 5–6 weeks of DIO. However, after this period the rate of weight gain in DC101-treated animals decreased significantly compared to the HFD controls (Figure 2A, DC101+HFD vs. HFD). This is consistent with our previous finding that VEGF-VEGFR2 pathway is critical for both angiogenesis and adipogenesis during *de novo* adipose tissue formation from preadipocytes [21]. We did not observe overt signs of toxicity in any of the experimental groups, similar to a lack of toxicity in tumor-bearing mice receiving DC101 for up to 75 days [26]. During the period of lower body weight gain, food intake in the DC101-treated group was significantly lower than in the control animals (Figure 2C). Therefore, we interrupted treatment to determine if the effect of DC101 of limiting fat expansion was reversible. The rate of weight gain resumed at a higher pace after cessation of anti-VEGFR2 treatment, eventually reaching the weights of untreated controls (Figure 2D), despite similar food intake during DC101 treatment and after cessation of treatment (2.35 vs 2.29 g/mouse/day).

## Discussion

Previous studies have established that fat tissue expansion is accompanied by an expansion of its supporting network of blood vessels [5], [20]. This expansion is likely driven both by the increased expression of angiogenic molecules (such as VEGF) during differentiation of progenitor cells into mature fat cells [15], and also by the local tissue hypoxia in fat tissue during obesity [27]. Whether the new endothelial cells required for this expansion are derived from the existing local vasculature, or if they are recruited from the bone marrow, has not been previously studied. In our model we found that BMDC contribution to blood vessels in fat tissue was marginal in mice fed either regular chow or high fat diet, but appeared increased in the fat tissue of aging mice. These findings suggest that BMDC contribution to fat tissue blood vessels likely reflects long-term replenishment by BMDC [28] rather than active recruitment for vasculogenesis. These data also suggest that new vessel formation during DIO occurs primarily by angiogenesis from pre-existing fat vessels.

We found that in mice treated with the VEGFR2-blocking antibody DC101, there was a significant decrease in food intake that coincided with the decrease in body weight gain. The reduced food intake undoubtedly contributed to the slower weight gain. However, after cessation of DC101 treatment, the animals maintained a similar level of reduced food intake, but were nevertheless able to increase their body weight until it was similar to that of control animals. This observation suggests that although food intake in DC101-treated mice was reduced compared to control mice, change in appetite alone does not completely account for the reduced body weight gain. We did not investigate how VEGFR2 blockade changes energy balance. Rather, based on our previous study showing that VEGFR2 inhibition inhibits *de novo* adipogenesis by restricting angiogenesis [21], and studies by others showing the importance of VEGF and VEGFR2 signaling in adipose tissue development [5], [29], [30], we hypothesized that inhibition of VEGFR2 in adipose tissue may have weight-reducing effects in diet-induced obesity. The results from our current study suggest several areas for future investigations, including the determination of how anti-angiogenic agents (such as DC101) affect different aspects of energy balance, what biological mechanism caused the DC101-induced reduction in food intake, as well as any possible linkage between adipose tissue angiogenesis and the regulation of appetite.

In this study we did not address the potential effects of VEGFR-inhibition on endocrine aspects of energy metabolism. However, a pervious study has reported that VEGF inhibition causes improved glucose tolerance in mice [31]. The molecular mechanism responsible for this outcome is unknown. The same study also showed that VEGF inhibition induced pruning of capillaries in the fat tissue of the treated mice.

Of interest, the body weight profile of mice with VEGFR2 blockade by DC101 treatment in this study is strikingly similar to data from mice genetically deficient for PlGF, a selective ligand for VEGFR1 [22]. In both studies the rate of body weight gain was initially unaffected by the anti-angiogenic treatment/genetic modification, then abruptly decreased after about 6 weeks. These results suggest that blocking angiogenesis may not be effective in preventing fat tissue growth during early stages of DIO, but may become important in later stages of expansion.

Anti-VEGFR1 treatment with MF1 had no effect on body weight throughout two months of treatment (Figure 2A, MF1+HFD vs. HFD). This finding is also consistent with the previous report that pharmaceutical neutralization of PlGF did not inhibit fat expansion in either diet-induced or genetic obesity [22]. As to why genetic deficiency in PlGF gave different results than pharmaceutical inhibition, one possible reason is difference in strain background. The PlGF knockout animals used by Linjen *et al* were of Swiss and 129SV background, while the animals used for antibody-blocking experiments in both our current study and the study by Linjen *et al* were of C57BL/6J background. Given the substantial body of literature showing that strain background has significant impact on metabolic phenotypes, it would not be surprising if the inhibition of the same genetic pathway could have divergent results in different strains.

VEGF-A and PlGF play important roles in recruitment of VEGFR1^+^ hematopoietic and VEGFR2^+^ endothelial BMDCs during new vessel formation in malignant tissues [32]. In our DIO models, we observed substantial infiltration of BMDCs into fat tissue (Figure 1D). However, incorporation of these cells into vessels was minimal, and we did not detect any conversion of BMDCs into adipocytes. It is likely that most of the BMDCs observed were infiltrating macrophages, known to accumulate in large numbers in the fat tissue of obese mice [10], [11]. Consistent with the lack of effect of VEGFR1 inhibition on DIO, we found that macrophage content in the fat was unchanged in the absence of VEGFR1 signaling (Tam et al., unpublished data).

Several studies using anti-vascular agents (which induce rapid endothelial cell death) in obese mice have shown immediate decreases in body weight [12], [13]. This effect is thought to be caused by damage to the existing blood vessels by induction of endothelial cell apoptosis. The results of our study suggest that pharmacologic blockade of VEGFR2—while unable to prevent DIO or reduce fat content—might be useful in limiting adipose tissue expansion in DIO. It should also be noted that another study using the anti-vascular agent TNP-470 in obese mice (both genetic and diet-induced) showed a slowing of body weight increase (rather than body weight decrease) that became evident after a “lag phase” of 2–3 weeks following initiation of treatment [14]. Both of these observations were similar to our results with VEGFR2 inhibition.

Two additional notes of caution are warranted regarding the implications of the results from our current study. Mammary fat pads were used in our bone marrow transplantation studies because they are the only fat tissue that can be observed, with intact circulation, by intra-vital microscopy. The depot-specific nature of fat tissue is now well established, and it must be emphasized that observations made in the mammary fat pad may not always be applicable to other fat depots. In addition, C57Bl/6J mice were chosen for the antibody blocking studies because the C57 strain is an established and well-characterized model of diet-induced obesity, whereas FVB mice were used in the microscopy studies because the transgenic mice with GFP expression were only available on FVB background. There are divergent reports in the literature regarding the effects of high fat diet on FVB mice – there are reports that FVB mice are resistant to diet-induced obesity [33], whereas others report that this strain does develop diet-induced obesity [34], albeit to a lesser extent than the C57 strain. Our data in FVB mice are consistent with the results by Martin *et al*, i.e. the high fat diet-fed FVB mice did develop diet-induced obesity. Nevertheless, since energy metabolism is known to be very sensitive to strain background, and results obtained using one strain generally cannot be assumed to apply to other strains without some experimental justification.

Several studies in recent years (including one from our laboratory) have explored the feasibility of fat tissue reduction by disrupting adipose tissue vasculature [12], [13], [14], [21]. The discovery that progenitors for white fat cells are derived from the mural cell compartment of adipose tissue vasculature [35] will undoubtedly increase interest in this approach. While reduction in body weight has been achieved in mice after anti-vascular or anti-angiogenic treatment, some caution is warranted in adapting this to clinical therapy, since the indiscriminant depletion of overall fat mass may lead to harmful lipodistrophic effects [36]. Any future therapeutic approach (such as anti-angiogenic treatments) that seeks to deplete fat mass must be carefully evaluated for this potential drawback.

Taken together, our results indicate that angiogenesis from local preexisting vasculature – and not the contribution of BMDCs – primarily sustains new vessel formation in fat tissue during DIO. Antiangiogenic treatment by antibody blockade of VEGFR2 but not of VEGFR1 restricted adipose tissue expansion. These data provide novel insight for the potential targeting of the fat vasculature to control DIO.
